# A Yin-Yang 1/*miR*-*30a* regulatory circuit modulates autophagy in pancreatic cancer cells

**DOI:** 10.1186/s12967-017-1308-3

**Published:** 2017-10-19

**Authors:** Chuang Yang, Jing-Jing Zhang, Yun-Peng Peng, Yi Zhu, Ling-Di Yin, Ji-Shu Wei, Wen-Tao Gao, Kui-Rong Jiang, Yi Miao

**Affiliations:** 1 Pancreas Center, First Affiliated Hospital of Nanjing Medical University, 300 Guangzhou Road, Nanjing, 210029 People’s Republic of China; 2 Pancreas Institute, Nanjing Medical University, 300 Guangzhou Road, Nanjing, 210029 People’s Republic of China

**Keywords:** Autophagy, YY1, *miR*-*30a*, Pancreatic cancer

## Abstract

**Background:**

Autophagy is a highly regulated biological process that mediates the degradation of intracellular components. It is required for tumor cell metabolism and homeostasis. Yin-Yang 1 (YY1) has been reported to be involved in autophagy in several carcinomas. However, its role in autophagy in pancreatic cancer, one of the deadliest human malignancies, is unknown. Here, we investigated the function of YY1 in pancreatic cancer cells autophagy and its mechanisms of action.

**Methods:**

The activity of cells undergoing autophagy was assessed using transmission electron microscopy, immunofluorescence, and Western blotting. A luciferase activity assay, real-time quantitative polymerase chain reaction (RT-qPCR), and chromatin immunoprecipitation (ChIP) were also used to identify putative downstream targets of YY1.

**Results:**

YY1 was confirmed to regulate autophagy in pancreatic cancer cells. It was found to directly regulate the expression of *miR*-*30a,* a known modulator of autophagy-associated genes. Furthermore, overexpression of *miR*-*30a* attenuated the pro-autophagic effects of YY1.

**Conclusions:**

Cumulatively, our data suggest that *miR*-*30a* acts in a feedback loop to modulate the pro-autophagic activities of YY1. Thus, autophagy in pancreatic cancer cells may be regulated, in part, by a tightly coordinated YY1/*miR*-*30a* regulatory circuit. These findings provide a potential druggable target for the development of treatments for pancreatic cancer.

## Background

Yin-Yang 1 (YY1) is a multifunctional zinc-finger transcription factor (also known as DELTA, NF-E1, UCRBP, and INO80S). It is ubiquitously expressed in all tissues and it is highly evolutionarily conserved from *Xenopus laevis* to humans [1]. The term “Yin-Yang” refers to its two opposing functions as either a transcriptional repressor or transcriptional activator during tumorigenesis. It has fundamental roles in embryogenesis, cell proliferation, and differentiation, which strongly suggests a potentially important function for YY1 during cancer development and progression [2]. YY1 typically binds genomic promoters to transactivate or repress gene expression. However, it can also indirectly activate or repress gene expression by interacting with histone modifiers and chromatin remodeling proteins [3]. In addition to regulating protein-coding genes, YY1 can also modulate the expression of noncoding genes, such as microRNAs (miRNAs) present in human cancer cells. For example, several recent studies have found that YY1 modulates tumorigenesis and the development of tumor cells by repressing the expression of numerous miRNAs, including *miR*-*9, miR*-*29, miR*-*146a,* and *miR*-*489* [4–7]. In addition, various types of cancers overexpress YY1 [8]. For instance, our previous study revealed that the expression of YY1 in pancreatic cancers is elevated compared to adjacent non-tumor tissues and normal pancreatic tissues [9].

Autophagy was first reported in 1962 by Ashford and Porter after they observed the accumulation of cytoplasmic components in glucagon-treated hepatic cells [10]. Autophagy has recently gained considerable attention by researchers. It is a highly regulated biological mechanism that facilitates the degradation of intracellular materials within autophagosomes by delivering them to lysosomes for bulk degradation. Autophagy is important for sustaining cell homeostasis and, likewise, is pivotal for tumor cell metabolism. Interestingly, many oncoproteins and oncosuppressors are involved in autophagy. They help cells survive a wide spectrum of stressful conditions [11]. Coincidentally, YY1 has also been reported to be required for autophagy [12]. Many recent studies have focused on the mechanisms of autophagy and its contributions to the development of malignancies, in addition to potential therapies. In pancreatic ductal adenocarcinoma, autophagy plays dual roles. It initially suppresses tumor initiation, but also supports tumor growth during later stages [13]. Furthermore, several studies have reported that autophagy protects pancreatic cancer cells from drugs and environmental stress [14, 15]. On the other hand, some investigators have reported that pancreatic cancer cells respond to various anticancer drugs by undergoing autophagy, which results in enhanced cytotoxicity [16, 17]. Although autophagy is vitally important during the development of pancreatic cancer, the mechanisms that activate autophagy remain largely unknown. In particular, the relationship between YY1 and autophagy in pancreatic cancer cells has not been reported.

In this study, we clearly demonstrate that YY1 modulates autophagy in pancreatic cancer cells by directly targeting *miR*-*30a*, an established regulator of the autophagy-associated genes, *ATG5* and Beclin 1 [18, 19]. Furthermore, *miR*-*30a* regulates the expression of YY1, suggesting a novel YY1/*miR*-*30a* regulatory circuit that controls autophagy in pancreatic cancer cells. Cumulatively, this study provides key insights that could aid the development of novel therapeutic strategies for treating pancreatic cancer.

## Methods

### Cell lines and cell culture

Six human pancreatic cancer cell lines (BxPC-3, CFPAC-1, Colo-357, MiaPaCa-2, PANC-1, and SW1990) were purchased from the Shanghai Cell Bank (Shanghai, China). The normal human pancreatic ductal cell line, HPNE, was purchased from the American Type Culture Collection (ATCC, USA). Cell lines that stably overexpress YY1 (BxPC-3 and PANC-1), cell lines in which YY1 is knocked down, and control cell lines were prepared and cultured as previously described [20]. Human pancreatic cancer cell lines were cultured in DMEM supplemented with 10% FBS, penicillin (100 U/mL), and streptomycin (100 μg/mL). The HPNE cell line was cultured according to the recommendations of the ATCC.

### qRT-PCR

Total RNA was extracted from different cell lines using Trizol reagent and reverse-transcribed into cDNA using the PrimeScript RT Master Mix according to the manufacturer’s instructions. For miRNA quantifications, miRNAs were extracted using the miRNeasy Mini Kit (Qiagen, China). Reverse-transcription was performed using the Mir-XTM miRNA First-Strand Synthesis Kit (Clontech, China) according to the manufacturer’s instructions. The mRNA expression levels were determined by qRT-PCR using the Step One Plus Real-Time PCR System (Applied Biosystems, USA) with the FastStart Universal SYBR Green Master according to the manufacturer’s instructions. The expression levels of the *YY1* mRNAs and *miR*-*30a* were normalized to *GAPDH* and *U6*, respectively, using the 2^−ΔΔCT^ method. Each qRT-PCR experiment was performed in triplicate and independently repeated three times.

### Western blotting

Pancreatic cancer cells or xenograft tumor tissues were lysed in ice-cold lysis buffer containing the following reagents: 50 mM Tris–HCl (pH 7.4), 1% NP-40, 150 mM NaCl, 1 mM EDTA, 1 mM PMSF, and a complete protease inhibitor cocktail (1 tablet per 10 mL, Roche Diagnostics GmbH, Mannheim, Germany). The total protein concentration was determined using a BCA Protein Assay kit (Beyotime Biotechnology, China). Western blotting was performed using standard methods. The following dilutions were used for each antibody: anti-YY1 (ab109228, Abcam, 1:3000), anti-Beclin 1 (#669922, R&D, 1:1000), anti-ATG5 (ab108327, Abcam, 1:3000), anti-P62 (ab109012, Abcam, 1:10,000), anti-LC3A/B (#12741, Cell Signaling, 1:1000), and anti-GAPDH (SAM1003, Sun Shine Bio, 1:5000). Each blot was independently repeated three times.

### Immunofluorescence

All reagents for fixation, washing, and blocking were purchased from Beyotime (Beyotime Biotechnology, China). Pancreatic cancer cells were washed with PBS three times for 5 min each and then fixed for 10 min. They were permeabilized with 0.1% Triton X-100 in PBS for 10 min. They were then blocked for 1 h at room temperature, followed by incubation with anti-LC3A/B antibody (#12741, Cell Signaling, 1:1000) at 4 °C overnight. After washing with PBS extensively the following day, cells were incubated with Cy3-Labeled Goat Anti-Rabbit IgG (Beyotime Biotechnology, China) for 3 h at room temperature. Chromatin was stained with DAPI for 10 min, and cells were observed under a fluorescence microscope (Nikon, Eclipse 80i, Japan).

### Transmission electron microscopy

Pancreatic cancer cells were washed with PBS twice and fixed in 2.5% glutaraldehyde in PBS at 4 °C overnight. Fixed cells were washed with PBS three times and post-fixed with 1% osmium acid in PBS for 3 h. After washing the cells three times with PBS, the samples were dehydrated in a graded ethanol series (30, 50, 70, 80, 90, 95, and 100%) for about 15–20 min for each incubation. Samples were then transferred to absolute acetone for 20 min. The samples were placed in a 1:1 mixture of absolute acetone, followed by incubation in a Spurr resin mixture for 1 h at room temperature. Samples were incubated in a 1:3 mixture of absolute acetone, followed by the final resin mixture for 3 h and a final Spurr resin mixture overnight. Finally, the specimens were placed in capsules containing embedding medium and heated to 70 °C for about 9 h. Sections 70 nm in thickness were generated using an ultramicrotome (Leica Ultracut R, Germany). Specimen sections were stained with uranyl acetate and lead citrate for 10 min each and observed using a JEM1230 (JEOL, Japan).

### *miR-30a* reagents and transfection

A *miR*-*30a* mimic, *miR*-*30a* inhibitor, and negative control mimic were purchased from Ribobio (Guangzhou, China). Lentiviruses used to knockdown or overexpress human *miR*-*30a*, in addition to negative control lentiviruses, were purchased from Hanbio (Shanghai, China). All transfections were carried out according to the manufacturer’s instructions.

### Luciferase assay

The luciferase assay was carried out as previously described [20]. Briefly, dual-luciferase reporter plasmids were constructed by iGeneBio (Guangzhou, China). Transfection of all plasmids was performed using the Lipofectamine 3000 Reagent. Luciferase activity was measured 48 h after transfection using the Dual-Luciferase Reporter Assay Kit (Promega,USA). The assay was repeated at least three times in independent experiments.

### ChIP assay

ChIP assays were performed as previously described [20] using the EZ ChIP Kit (Merck Millipore, Germany) according to the manufacturer’s instructions. Briefly, cross-linked chromatin was sonicated into fragments ranging from 100 to 1000 base pairs. The chromatin was then immunoprecipitated using a ChIP-grade anti-YY1 antibody (ab12132, Abcam).

### PANC-1 xenograft tumor model

All animal research procedures adhered to the guidelines of *The Care and Use of Laboratory Animals* published by the National Institutes of Health and were approved by the Laboratory of Animal Care and Use Committee of Nanjing Medical University. Four-week-old female nude mice (BALB/cA-nu) were purchased from the Model Animal Research Center of Nanjing University. Twenty mice were randomly divided into four groups. To establish a xenograft subcutaneous implant tumor model, PANC-1-YY1-knockdown and vector control cells, PANC-1-miR-30a-knockdown and vector control cells (1.5 × 10^6^ cells/100 μL per mouse) were injected subcutaneously. The mice were euthanized after 4 weeks, and tumor xenografts were resected.

### Statistical analysis

All data were representative of at least three independent experiments. Statistical analysis was performed using the SPSS software (Version 22.0). Quantitative data are presented as mean ± SD. All statistical tests were two-tailed exact tests with a p < 0.05 considered significant.

## Results

### YY1 is implicated in autophagy in pancreatic cancer cells

**Fig. 1:**
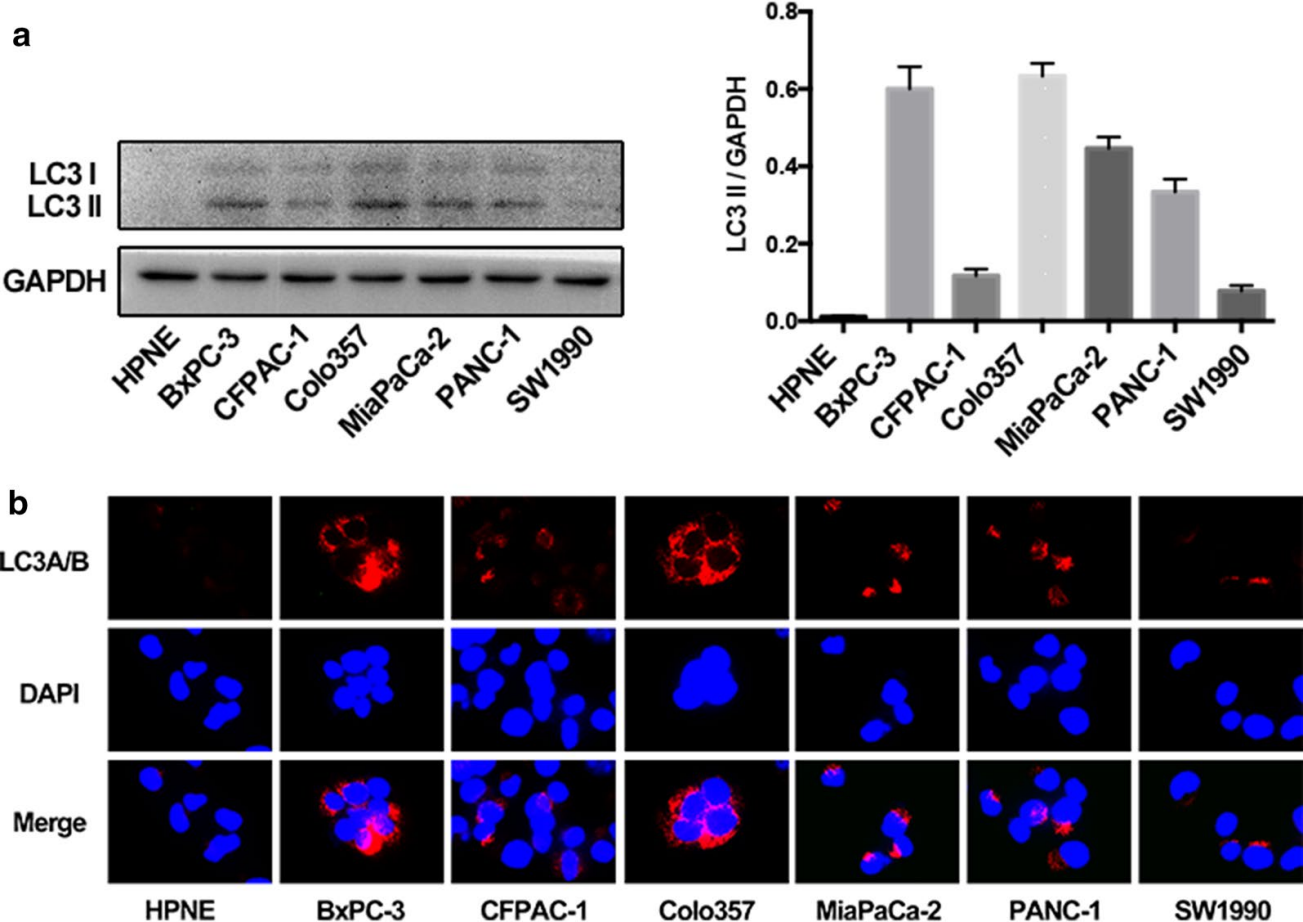
The level of autophagy in pancreatic cancer cells. **a** The expression of LC3 II were examined by Western blotting in pancreatic HPNE cells and six pancreatic cancer cell lines (BxPC-3, CFPAC-1, Colo357, MiaPaCa-2, PANC-1, and SW1990) cultured under normal growth conditions. Intensity quantified by image J were given in the histogram. **b** The expression of LC3 II in pancreatic cancer cell lines and HPNE cells was assayed using Immunofluorescence. LC3 subcellular localization is shown in red and nuclear DAPI staining is shown in blue. Results are representative of three independent experiments

**Fig. 2:**
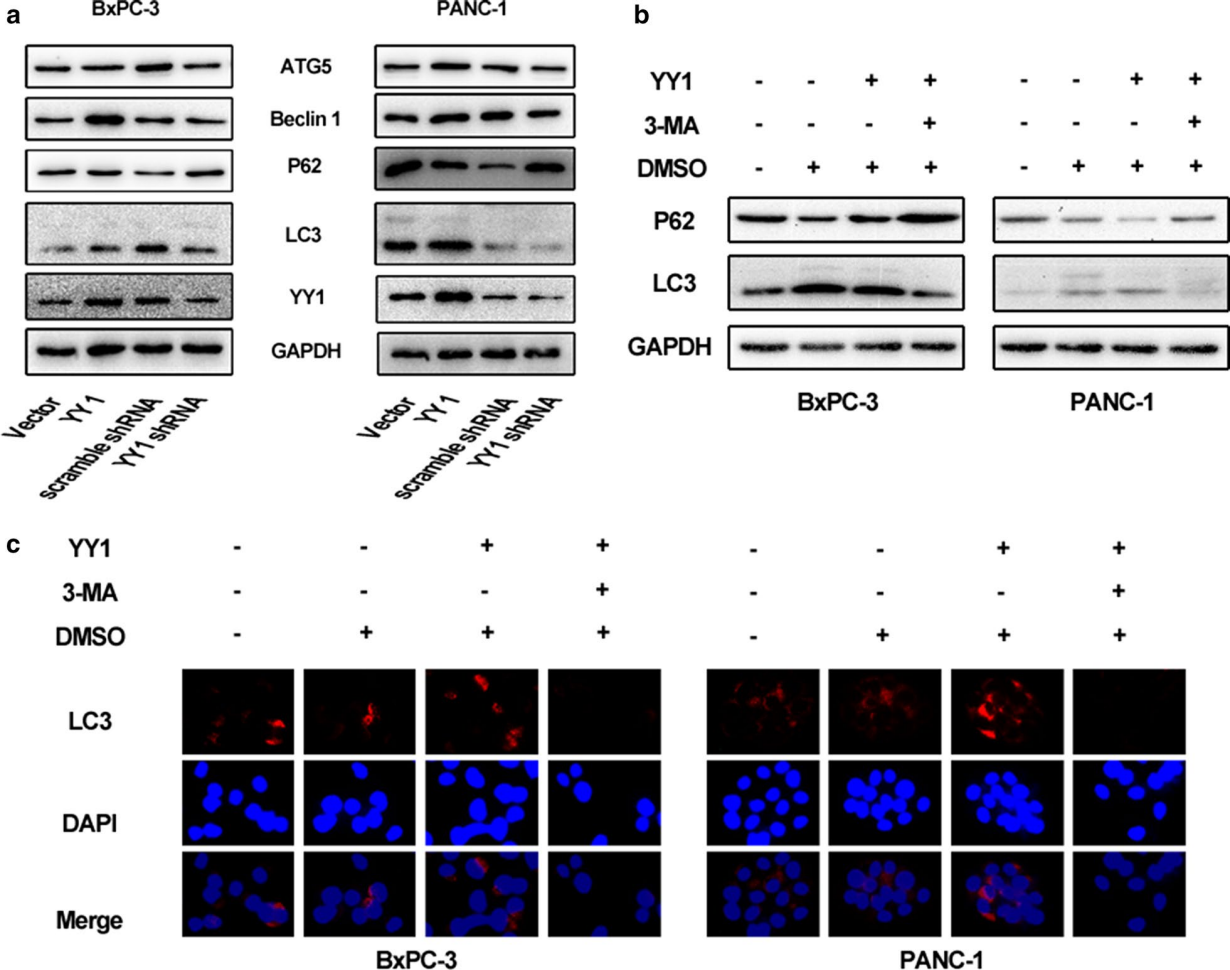
YY1 plays roles in autophagy in pancreatic cancer cells. **a** The expression of the autophagy-associated proteins, LC3, p62, ATG5, and Beclin1, in YY1-overexpressing or YY1 knockdown BXPC-3 and PANC-1 cells assayed by Western blotting. “YY1” indicates YY1-overexpressing cells, “Vector” indicates cells transfected with a control vector, “YY1 shRNA” indicates YY1 knockdown cells, and “Scramble shRNA” indicates cells transfected with a vector expressing Scramble shRNA. **b** YY1-overexpressing or YY1 knockdown BXPC-3 and PANC-1 cells were treated with 10 mM 3-MA for 8 h. Untreated cells and the vehicle group treated with DMSO were used as controls. Autophagy levels were then assayed using Western blotting. **c** LC3 levels were assayed using Immunofluorescence. Results are representative of at least three independent experiments

**Fig. 3:**
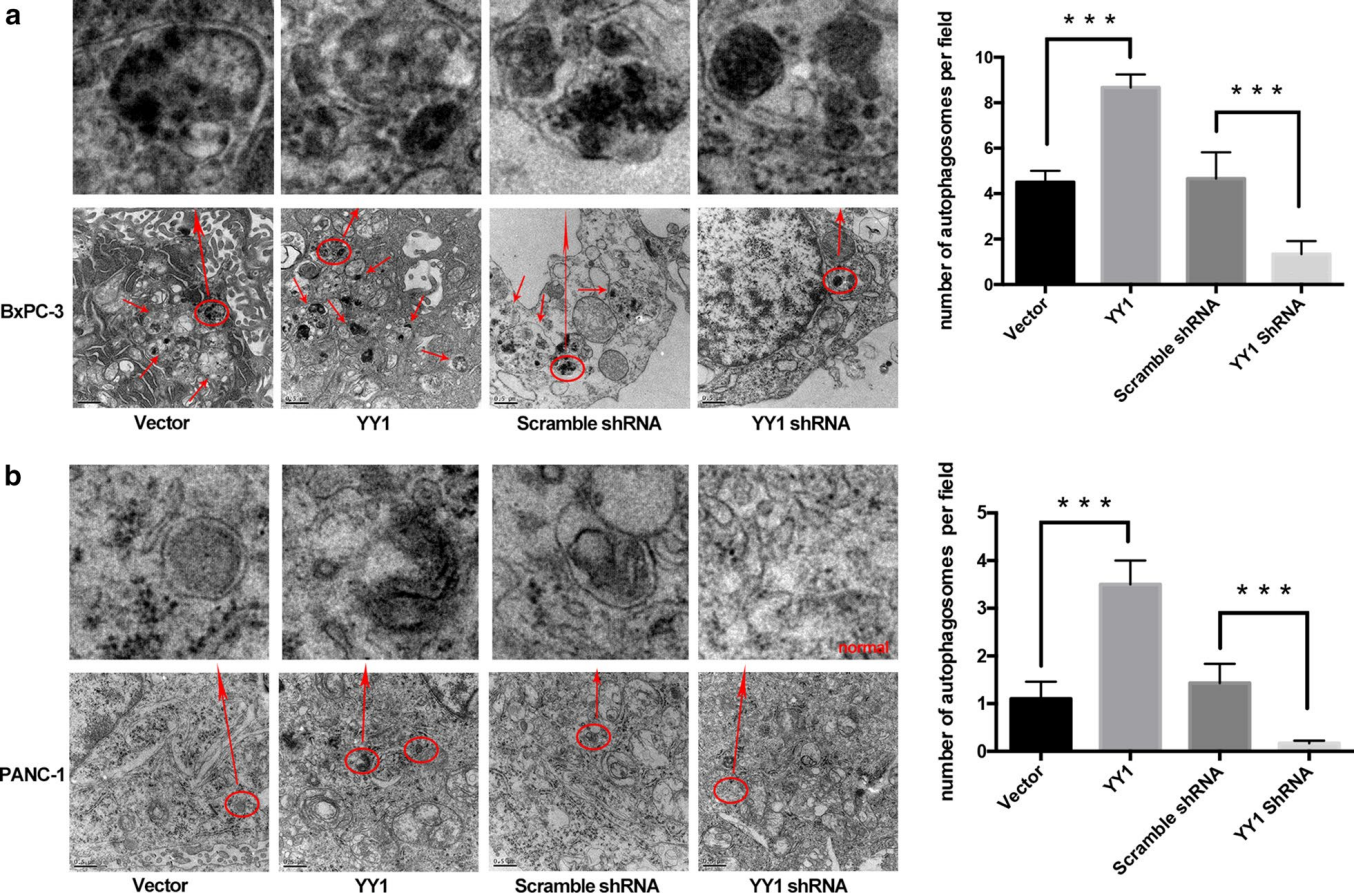
Autophagosomes detected by transmission electron microscopy. **a** Transmission electron microscopy shows autophagosomes (red circles) in YY1-overexpressing or YY1 knockdown BXPC-3. Images were captured at ×20,000 magnification. The representative autophagosome was standalone enlarged above. The number of autophagosomes per field was acounted in the histogram. ***p < 0.001. **b** Transmission electron microscopy shows autophagosomes (red circles) in YY1-overexpressing or YY1 knockdown PANC-1. Images were captured at ×20,000 magnification. The representative autophagosomes were standalone enlarged above. The number of autophagosomes per field was accounted in the histogram. ***p < 0.001

During autophagosome formation, the cytosolic microtubule-associated protein 1 light chain 3 (LC3)-I is converted into LC3-II, it is phosphatidylethanolamine- conjugated form. LC3II then targets autophagic membranes [21]. To determine the state of autophagy in pancreatic cancer cells, Western blotting was performed for LC3 II in six pancreatic cancer cell lines, and results were compared to those of the control cell line, HPNE (Fig. 1a). Autophagy was clearly elevated in pancreatic cancer cells, which was reflected by the increased in LC3II. Immunofluorescence further confirmed it (Fig. 1b). Next, to investigate the potential role of YY1 in autophagy, we either overexpressed or silenced YY1 in BxPC-3 and PANC-1 cells. We found that YY1 overexpression increased LC3II levels, while YY1 knockdown decreased LC3II levels. Furthermore, other autophagy-related genes, *ATG5* and Beclin-1 (the mammalian homologue of yeast ATG6), displayed similar expression dynamics as LC3II (Fig. 2a). In addition, Western blotting and immunofluorescence showed that, compared to the YY1-treated group, autophagy was significantly reduced following treatment with a combination of YY1 and the autophagy inhibitor, 3-methyladenine (3-MA), suggesting that YY1-induced autophagy is inhibited by 3-MA (Fig. 2b, c). To better assess autophagy, we observed autophagosome formation and double-membrane autophagic vacuoles by transmission electron microscopy in both BxPC-3 and PANC-1 cells (Fig. 3a, b). From these micrographs, we found that overexpression of YY1 increased the number of autophagosomes, while YY1 knockdown decreased the abundance of autophagosomes. Taken together, YY1 likely plays roles in autophagy in pancreatic cancer cells.

### YY1 binds upstream of the *miR-30a* genomic loci and regulates its expression

**Fig. 4:**
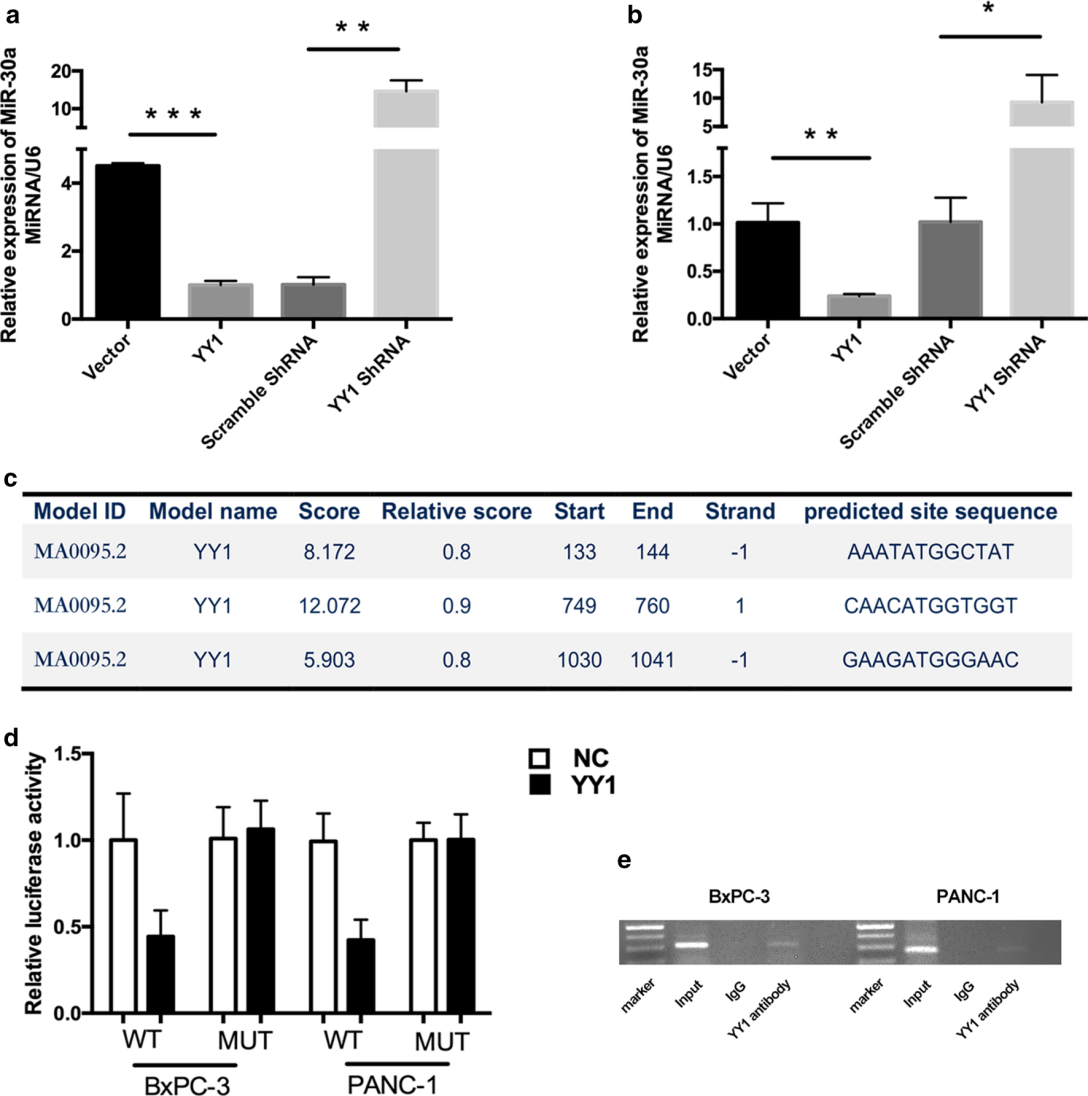
YY1 binds upstream of the miR-30a genomic loci and regulates its expression. **a** qRT-PCR analysis for *miR*-*30a* expression levels in YY1-overexpressing or YY1 knockdown BXPC-3 cells. Results are representative of three independent experiments and are presented as the mean ± SD (bars). *p < 0.05, **p < 0.01, ***p < 0.001. **b** qRT-PCR analysis for *miR*-*30a* expression levels in YY1-overexpressing or YY1 knockdown PANC-1 cells. **c** Three putative YY1 binding sites upstream of the *miR*-*30a* genomic locus predicted using JASPAR bioinformatics software. **d** A luciferase activity assay was used to determine the effect of YY1 on the expression of a luciferase reporter driven by a *miR*-*30a* 3′UTR fragment with or without YY1-specific binding sites in BXPC-3 and PANC-1 cells. **e** The binding of YY1 to the *miR*-*30a* genomic locus was determined by ChIP

Synergies between YY1 and miRNAs have recently been reported in various cellular processes [5, 12, 22]. Therefore, it is possible that autophagy-related miRNAs might be directly regulated by YY1. Previously, we found that YY1 regulates the expression of ATG5 and Beclin 1 proteins (Fig. 2a). Since ATG5 and Beclin 1 are both known to be direct targets of *miR*-*30a*, [19, 23] we hypothesized that YY1 and *miR*-*30a* might form a functional regulatory circuit that modulates autophagy in pancreatic cancer cells. Using an RT-PCR assay, *miR*-*30a* levels were found to decrease when YY1 was overexpressed and increase when YY1 was knocked down in both BxPC-3 and PANC-1 cells (Fig. 4a, b). This suggested that *miR*-*30a* might negatively be regulated by YY1. A bioinformatics prediction algorithm (http://jaspar.binf.ku.dk) revealed three putative YY1 binding sites upstream of the *miR*-*30a* genomic loci (Fig. 4c). Using the predicted site sequence that displayed the highest score, we constructed luciferase reporter vectors and performed a dual-luciferase reporter assay. The results showed that overexpression of YY1 significantly decreased luciferase activity for the *miR*-*30a* 3′UTR compared to the negative control. In contrast, a mutated version of the *miR*-*30a* 3′UTR showed unchanged luciferase activity, demonstrating direct regulatory control of miR-30a (Fig. 4d). To map the YY1 binding sites in the *miR*-*30a* promoter under physiological conditions, a ChIP assay was performed. The results revealed that YY1 directly bound the promoter of *miR*-*30a* and repressed its expression in BxPC-3 and PANC-1 cells (Fig. 4e). Taken together, these experiments verify *miR*-*30a* as a direct target of YY1.

### Ectopic expression of *miR-30a* decreases YY1-induced autophagy

**Fig. 5:**
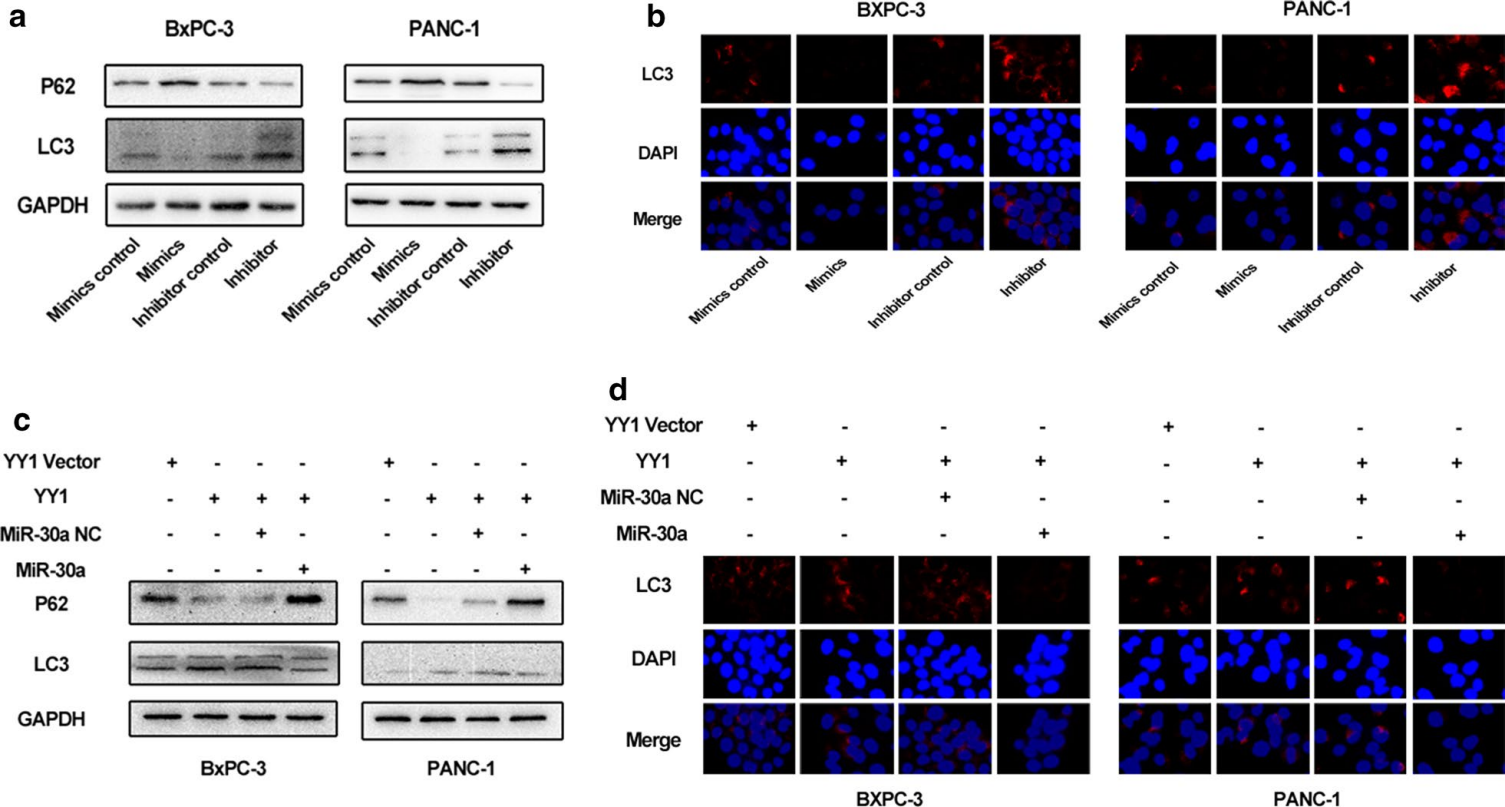
Ectopic expression of miR-30a decreases YY1-induced autophagy. **a** Western blotting was performed to quantify the expression levels of LC3I/II and p62 in BXPC-3 and PANC-1 cells after transfection with a *miR*-*30a* mimic, a *miR*-*30a* inhibitor, or a negative control for 48 h. **b** LC3A/B in BXPC-3 and PANC-1 cells after transfection with a *miR*-*30a* mimic, a *miR*-*30a* inhibitor, or a negative control for 48 h were detected by Immunofluorescence. **c** The expression of LC3I/II and p62 in BXPC-3 and PANC-1 cells transfected with YY1-overexpressing lentiviruses or *miR*-*30a* mimics were quantified using Western blotting. **d** The expression of LC3A/B in BXPC-3 and PANC-1 cells transfected with YY1-overexpressing lentiviruses or *miR*-*30a* mimics were quantified using Immunofluorescence. Results are representative of at least three independent experiments

Since *miR*-*30a* was found to be a direct target of YY1, we next investigated whether *miR*-*30a* mediated the autophagy-related effects of YY1. We first manipulated the expression levels of *miR*-*30a* in both BxPC-3 and PANC-1 cells using a *miR*-*30a* mimic, a *miR*-*30a* inhibitor, and a control miRNA. Using Western blotting and immunofluorescence, we found that *miR*-*30a* negatively regulated autophagy in pancreatic cancer cells (Fig. 5a, b). Furthermore, we performed a rescue assay in BxPC-3 and PANC-1 cells consisting of a treatment that combined YY1-overexpressing lentiviruses and *miR*-*30a* mimics. As expected, overexpression of *miR*-*30a* attenuated the pro-autophagic effects of YY1 (Fig. 5c, d), suggesting that *miR*-*30a* is indeed a functional target of YY1.

### *miR-30a* acts in a feedback loop to inhibit YY1

**Fig. 6:**
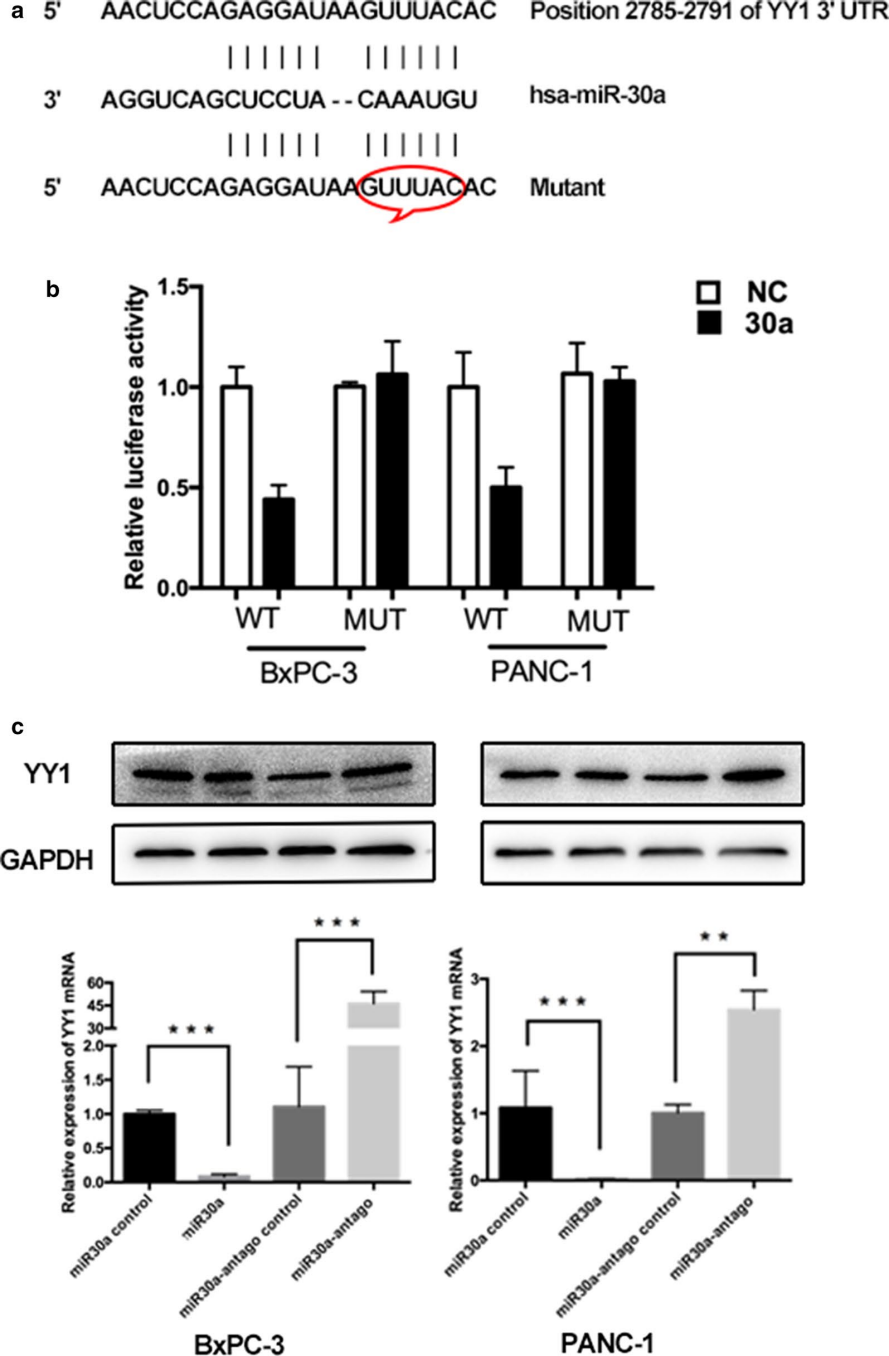
miR-30a acts in a feedback loop to inhibit YY1. **a** Predicted binding sites for *miR*-*30a* in the YY1 3′UTR. The engineered mutant sequence is also shown. **b** The luciferase activities of BXPC-3 and PANC-1 cells were measured after co-transfection with the indicated YY1 3′UTR constructs and *miR*-*30a* for 24 h. **c** Western blot and qRT-PCR analysis of YY1 protein or mRNA levels in BXPC-3 and PANC-1 cells transfected with *miR*-*30a*-overexpressing or -inhibiting lentivirus. **p < 0.01, ***p < 0.001

Since YY1 appears to promote autophagy in pancreatic cancer cells by directly targeting *miR*-*30a*, we next explored whether *miR*-*30a* modulates YY1 expression, since this type of feedback regulation is commonly observed between transcription factors and miRNAs (such as the Pitx3/*miR*-*133b* feedback circuit in midbrain dopamine neurons) [24, 25]. Using various bioinformatics tools, such as Targetscan [26] and RNA22-HAS [27], we found a putative *miR*-*30a* binding site in the 3′UTR of *YY1* (Fig. 6a). To test whether the predicted *YY1* 3′UTR binding sites reflect direct regulation by *miR*-*30a*, we inserted these sequences downstream of a luciferase reporter gene. We found that a decrease in luciferase activity occurred in the presence of wild type YY1. In contrast, this luciferase inhibition was rescued by mutating the *YY1* 3′UTR sequences that were predicted to be the *miR*-*30a* binding sites (Fig. 6b). We further tested the effect of *miR*-*30a* on YY1 mRNA and protein expression using RT-PCR and Western blotting, respectively. This revealed that the expression levels of YY1 were elevated in cells transfected with the *miR*-*30a* inhibitor lentivirus and decreased in cells transfected with the *miR*-*30a* overexpressing lentivirus (Fig. 6c). Cumulatively, these results suggest that *miR*-*30a* acts in a feedback loop to inhibit YY1, thereby regulating autophagy in pancreatic cancer cells.

### YY1/miR30a regulates autophagy in pancreatic xenograft tumors

**Fig. 7:**
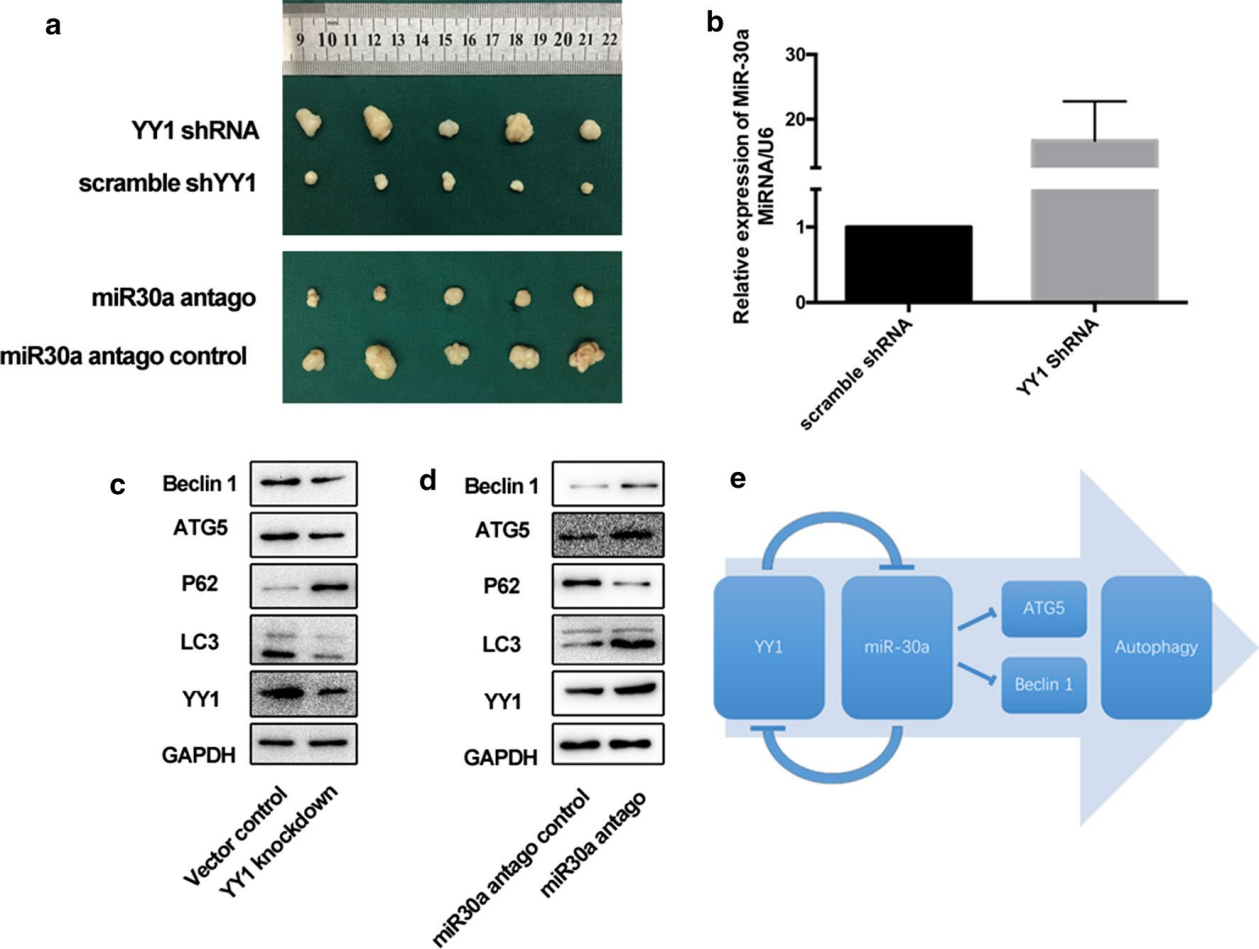
YY1/miR30a regulates autophagy in pancreatic xenograft tumors. **a** The in vivo tumorigenicity of PANC-1 cells with or without YY1/miR30a expression was determined using a nude mouse xenograft assay. Results are representative of three independent experiments. **b** qRT-PCR analysis of miR30a expression in pancreatic xenograft tumors. **c** ATG5 and Beclin-1 expression and autophagy activity in the YY1-knockdown xenograft samples assayed by Western blotting. Results are representative of three independent experiments. **d** ATG5 and Beclin-1 expression and autophagy activity in the miR30a-knockdown xenograft samples assayed by Western blotting. Results are representative of three independent experiments. **e** A model of the YY1/*miR*-*30a* regulatory interaction during autophagy in pancreatic cancer cells

To assess the effects of YY1-induced autophagy in vivo, we implanted xenograft tumors with alternatively varying levels of both YY1 and miR-30a. The mice were euthanized after 4 weeks, and tumor xenografts were resected and photographed (Fig. 7a). The results showed that knockdown of YY1 increased the expression of miR30a (Fig. 7b). We also quantified ATG5 and Beclin-1 expression levels and overall autophagy activity in the xenograft samples (Fig. 7c). Consistent with the in vitro results, knockdown of YY1 inhibited autophagy through miR30a. On the other hand, when nude mice were subcutaneously injected with PANC-1 cells that had been transfected with either a lentiviral vector that inhibited miR30a or a control lentiviral vector, we found that knockdown of miR30a could also increased the expression of YY1 and autophagy activity (Fig. 7d).

## Discussion

This study investigated potential roles for YY1 in autophagy in pancreatic cancer cells. We uncovered the existence of a novel YY1/*miR*-*30a* regulatory circuit that modulates autophagy in BxPC-3 and PANC-1 cells. YY1 promotes autophagy in pancreatic cancer cells by suppressing the expression of *miR*-*30a*, which modulates the autophagy-associated genes, *ATG5* and Beclin 1. Furthermore, YY1 is also targeted by *miR*-*30a* via a negative feedback loop (Fig. 7e).

Based on the available literature, YY1 has diverse and complex biological functions. However, research regarding its roles during autophagy is lacking. In pancreatic cancer, autophagy is upregulated during later stages of the disease in a cell-autonomous fashion, and it is required for continued tumor growth in vitro and in vivo [14]. Understanding the role of autophagy in pancreatic cancer pathogenesis is critical. Therefore, we explored the mechanisms of autophagy regulation in pancreatic cancer. In this study, we implicated YY1 in the regulation of autophagy in pancreatic cancer cells. Combined with our previous work, YY1 appears to play critical roles in pancreatic cancer—not only in tumor cell growth, progression, and invasion, but also in autophagy [9]. In our previous study, we showed that YY1 promotes apoptosis and plays a tumor-suppressive role in pancreatic cancer cells [20]. In the present study, we find that YY1 also promotes autophagy and attenuates pancreatic cancer growth. Interestingly, these results seem to contradict a previous study that showed that inhibition of autophagy suppresses proliferation of BxPC-3 and PANC-1 cells [28]. It is possible that these tumor-suppressive effects mainly depend on YY1-induced apoptosis. In addition, many studies have shown that autophagy induces apoptosis, and autophagy-associated proteins (such as ATG5, ATG12, and Beclin1) help initiate apoptosis [29, 30]. Since YY1 has been observed to promote both apoptosis and autophagy in pancreatic cancer, the effect of suppressing tumor growth clearly requires further investigation.

Transcription factors and microRNAs can act in cooperation to regulate target gene expression via feed forward or feedback loops. These regulatory interactions play critical roles in many biological processes and they have been extensively studied over the years [24, 31]. Our findings support the existence of reciprocal regulation between YY1 and a miRNA. Interestingly, the inhibitory feedback interaction between YY1 and *miR*-*30a* is similar to the observations of Lu and his team [32], who demonstrated the existence of a novel YY1/*miR*-*1* regulatory circuit during skeletal myogenesis. Feedback loops of this type may be advantageous to cells because it allows the system to remain reversible so that the system will be more stable.

## Conclusions

In summary, we have identified a novel signaling circuit consisting of YY1 and *miR*-*30a*, which is required for regulating autophagy in pancreatic cancer cell lines. This research sheds new light on mechanisms critical for promote tumorigenesis and tumor progression in pancreatic cancer. Because the pathways downstream of YY1 are highly redundant, other currently unknown mechanisms may be involved in YY1-mediated autophagy. The interaction between YY1 and *miR*-*30a* illustrates the importance of feedback control in autophagy, and this circuit may constitute a valuable diagnostic and therapeutic target for treating pancreatic cancer.

## Abbreviations

YY1: Yin-Yang 1
RT-qPCR: real-time quantitative polymerase chain reaction
ChIP: chromatin immunoprecipitation
DMEM: Dulbecco’s modified Eagle’s medium
FBS: fetal calf serum
NP-40: Nonidet P 40
EDTA: ethylene diamine tetraacetie acid
PMSF: phenylmethanesulfonyl fluoride
BCA: bicinchoninic acid
DAPI: 4′,6-diamidino-2-phenylindole
DMSO: dimethyl sulphoxide
